# Can EEG‐Neurofeedback Training Enhance Effective Connectivity in People With Chronic Secondary Musculoskeletal Pain? A Secondary Analysis of a Feasibility Randomized Controlled Clinical Trial

**DOI:** 10.1002/brb3.70541

**Published:** 2025-05-28

**Authors:** Jerin Mathew, Divya Bharatkumar Adhia, Mark Llewellyn Smith, Dirk De Ridder, Ramakrishnan Mani

**Affiliations:** ^1^ Centre for Health, Activity, and Rehabilitation Research, School of Physiotherapy University of Otago Dunedin New Zealand; ^2^ Department of Anatomy, School of Biomedical Sciences University of Otago Dunedin New Zealand; ^3^ Pain@Otago Research Theme University of Otago Dunedin New Zealand; ^4^ Division of Neurosurgery, Department of Surgical Sciences, Dunedin School of Medicine University of Otago Dunedin New Zealand; ^5^ Neurofeedback Therapy Services New York USA

**Keywords:** clinical trial, EEG, effective connectivity, infraslow fluctuations, knee osteoarthritis, neurofeedback, persistent pain

## Abstract

**Introduction:**

Persistent musculoskeletal pain is associated with altered functional and effective connectivity (EC) between cortical regions involved in pain processing. Especially, disruptions in the infraslow fluctuation (ISF) frequency band can contribute to pain persistence. ISF electroencephalography‐neurofeedback (EEG‐NF) has emerged as a potential non‐invasive neuromodulatory intervention targeting cortical brain regions to restore balance and modulate pain‐related pathways. However, limited research explores its effect on EC, a measure of directional information flow critical to pain experience and modulation.

**Methods:**

A secondary analysis was performed using data from a randomized, double‐blind, sham‐controlled feasibility clinical trial. Participants with chronic painful knee osteoarthritis (OA) were randomized to receive either ISF‐NF or sham‐NF. Nine neurofeedback sessions targeted the pregenual anterior cingulate cortex (pgACC), dorsal anterior cingulate cortex (dACC), and bilateral primary somatosensory cortex (SSC: S1Lt & S1Rt). EEG data was collected at baseline and post‐intervention. Granger causality was used to measure EC changes, and between‐group statistical analyses were conducted with adjustments for multiple comparisons.

**Results:**

Twenty‐one participants (mean age: 61.7 ± 7.6 years; 62% female) completed the study. ISF‐NF training significantly improved EC between pgACC and dACC, pgACC and SSC, and other targeted regions, while reducing EC from S1Rt to dACC. Changes were observed predominantly in the ISF frequency band, indicating enhanced cortical communication and modulation of pain pathways.

**Conclusion:**

ISF‐NF training enhanced EC in cortical regions implicated in pain processing, supporting its potential as a neuromodulatory intervention for chronic musculoskeletal pain. Further trials are needed to confirm clinical efficacy and optimize protocol designs.

## Graphical abstract

Infraslow neurofeedback training improved effective connectivity in cortical regions involved in pain processing, highlighting its potential as a non‐invasive neuromodulatory intervention for chronic musculoskeletal pain. However, further research is required to validate its clinical efficacy and refine protocol designs to optimize its application in managing persistent pain conditions.

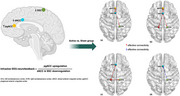

## 1 Introduction

Chronic pain is a complex condition that entails significant interactions among multiple brain regions responsible for pain processing and modulation (Mathew et al. 2024a; De Ridder et al. 2022; Henderson et al. 2013; Ploner et al. 2017). Chronic pain is hypothesized to involve dysregulation of cortical neuronal activity and altered connectivity between ascending and descending pain‐modulatory brain regions, as observed in conditions such as neuropathic pain, fibromyalgia, and knee osteoarthritis (OA) (Vanneste and De Ridder 2021; De Ridder et al. 2021; Mathew et al. 2024a). Neuroimaging studies have demonstrated activity and connectivity alterations in these regions, particularly involved in somatosensory processing (e.g., somatosensory cortex, SSC), affective‐motivational responses (e.g., dorsal anterior cingulate cortex, dACC; anterior insular cortex), and descending pain modulation (e.g., pregenual anterior cingulate cortex, pgACC) (De Ridder et al. 2021; De Ridder et al. 2022; Barroso et al. 2021; Yang and Chang 2019; Lewis et al. 2018; Brandl et al. 2022; Bushnell et al. 2013; Price 2000; Fields 2004). Moreover, changes in functional connectivity (the communication between two distinct brain regions) among brain regions associated with pain perception have been observed in various chronic pain conditions and are considered a factor in pain persistence (Motoyama et al. 2019; Necka et al. 2019; Thorp et al. 2018; Kaplan et al. 2019; Spisak et al. 2020; Fallon et al. 2016).

Non‐invasive neuromodulation has been suggested as a possible intervention to regulate activity and connectivity among cortical regions involved in pain processing for chronic pain management (Meeker et al. 2020; Roy et al. 2020; Adhia et al. 2023; Yang et al. 2024). Electroencephalography Neurofeedback (EEG‐NF) is a brain‐computer interface technique that can be used to enhance or suppress brain activity in specific regions associated with various disease conditions (Mathew J. 2025; Roy et al. 2020; Enriquez‐Geppert et al. 2017; Marzbani et al. 2016; Sitaram et al. 2017). Studies have shown that EEG‐NF effectively reduces pain associated with various chronic pain conditions, as demonstrated by validated outcome measures (Hesam‐Shariati et al. 2022; Adhia et al. 2023; Mathew et al. 2022). However, understanding its underlying mechanisms requires investigating whether EEG‐NF training induces changes in brain activity and connectivity (Hesam—Shariati et al. 2021). Previous research has reported changes in activity and functional connectivity within targeted brain regions following EEG‐NF training (Adhia et al. 2023; Mathew et al. 2024b). While it is well‐established that EEG‐NF can alter functional connectivity between cortical regions, the direction of these connections (effective connectivity; EC) and their strength remain understudied. Previous work has shown that EEG‐NF can enhance functional connectivity between the SSC and pgACC in distinct frequency bands (Mathew et al. 2024b). However, the directionality of these connections (EC) remains unclear. Since altered connectivity between pain‐related brain regions has been linked to pain perception (Vanneste and De Ridder 2021; Necka et al. 2019), exploring whether EEG‐NF can modulate these connections could provide further understanding into its therapeutic mechanisms.

While functional connectivity reflects statistical dependencies between brain regions, EC captures the directionality of these interactions (Friston 2011), which is crucial for understanding causal influences in pain networks. Isolated effective coherence (iCoh) (also known as directed functional connectivity/EC) is a measure that demonstrates the causal information flow between distinct brain regions (Pascual‐Marqui et al. 2014). Alterations in iCoh/EC have been observed in individuals with chronic pain (Vanneste and De Ridder 2021; Ferdek et al. 2019; Spisák et al. 2017; Wu et al. 2013). For instance, a previous study reported a reduction in theta‐band EC from the pgACC to the bilateral SSC and vice versa (Vanneste and De Ridder 2021). This may imply that the cortical regions associated with the descending pain inhibitory pathway are inhibited by the overactivation of the SSC, potentially contributing to the persistence of pain (Vanneste and De Ridder 2021). Similarly, research on chronic low back pain has reported altered resting state EC within the cingulo‐frontal‐parietal cognitive attention networks (Mao et al. 2022). Comparable findings have also been reported in conditions like endometriosis, where heightened beta‐band EC has been observed from the left dorsolateral prefrontal cortex to the SSC, as well as from the SSC to the orbitofrontal and right temporal cortices (Ferdek et al. 2019).

iCoh is a dependable and reliable measure of brain EC changes following neuromodulatory approaches targeting brain regions to improve pain experience (Adhia et al. 2023; Cao et al. 2022). However, few studies have examined changes in connectivity and iCoh following EEG‐NF, particularly between key pain‐related cortical regions. Only one study has investigated the EC following EEG‐NF training in people with chronic low back pain. The findings indicated that infraslow fluctuation (ISF)‐NF training targeting the pgACC can improve pain by modulating EC between the pgACC and SSC. Moreover, the study also demonstrated an association between EC changes from pgACC to SSC and pain severity (Adhia et al. 2022). Therefore, this exploratory secondary analysis investigates whether ISF EEG‐NF training can modify EC between the pgACC, SSC, and dACC in individuals with knee osteoarthritis (OA)—a chronic secondary musculoskeletal pain condition associated with significant alterations in cortical brain regions involved in pain processing (Mathew et al. 2024a).

## 2 Methods

### 2.1 Trial Registration and Ethical Approval

This study received ethical approval from the Health & Disability Ethics Committee (HDEC), New Zealand (19CEN182), and was registered with the Australian New Zealand Clinical Trials Registry (ACTRN12620000273987). A detailed version of the study protocol and feasibility outcomes has been published elsewhere (Mathew et al. 2022; Mathew et al. 2020). Written informed consent was obtained from all participants prior to their enrollment in the study. Consolidated Standards of Reporting Trials (CONSORT) (extension for randomized pilot and feasibility trials) guidelines were used to report the undertaken methodology (Eldridge et al. 2016). The intervention was detailed following the Template for Intervention Description and Replication (TIDieR) framework (Hoffmann et al. 2014).

### 2.2 Study Design

A secondary analysis was performed using data from a randomized, double‐blind, sham‐controlled feasibility clinical trial that was conducted to investigate the feasibility and safety of a source‐localized EEG‐NF for the management of chronic pain secondary to knee OA. The study aimed to modulate ISF electrical activity in the three cortical regions associated with pain processing, namely the dorsal anterior cingulate cortex (dACC), a cortical hub processing the affective/motivational component of pain (Rainville 2002; Bushnell et al. 2013; Price 2000; Vogt and Sikes 2000); the primary somatosensory cortex (S1), a proxy for processing the discriminative/sensory component of pain (Gingold et al. 1991; Craig 2002; Bushnell et al. 1999), and the pregenual anterior cingulate cortex (pgACC); part of the cortical pain inhibitory pathway (Kwon et al. 2014; Ossipov et al. 2014; Vanneste et al. 2017).

### 2.3 Participant Characteristics

#### 2.3.1 Inclusion Criteria

Individuals between the ages of 44 and 75 years with a clinical diagnosis of knee OA and experiencing pain (rated ≥4 on an 11‐point numerical scale) for at least three months were eligible for participation in the study (Mathew et al. 2022; Mathew et al. 2024b; Mathew et al. 2020).

#### 2.3.2 Exclusion Criteria

Exclusion criteria included recent or upcoming surgery, recent steroid use, centrally acting medications, neurological disorders, knee soft tissue injuries, cognitive impairments, language barriers, and pregnancy or postpartum status (≤ six months) (Mathew et al. 2022; Mathew et al. 2024b; Mathew et al. 2020).

### 2.4 Randomization and Concealment of Allocation

Participants were assigned to either the ISF‐NF (active) or sham NF (control) group through a block randomization method using an open‐access program. The randomization process was managed by a research administrator. Group assignments remained concealed until the initial assessment was completed. Neither the participants nor the outcome assessor were aware of the group allocation (Mathew et al. 2022; Mathew et al. 2024b; Mathew et al. 2020).

### 2.5 Interventions

Eligible participants took part in nine NF sessions (30 min each; three sessions per week) and two 90 min assessment sessions at baseline (T0) and post‐intervention (T1). The experimental procedures and reporting of NF adhered to the Consensus on the Reporting and Experimental Design of Clinical and Cognitive‐Behavioral Neurofeedback Studies (CRED‐nf checklist—mandatory items) (Ros et al. 2020).

The NF was administered using a 21‐channel DC‐coupled amplifier (BrainMaster Technologies Inc., Bedford, Ohio, United States of America) connected to a high‐end laptop with BrainAvatar 4.0 software installed for real‐time signal processing and for providing feedback. Each participant was instructed to wear the Comby EEG lead cap equipped with Ag/AgCl sensors, with reference electrodes placed on the mastoids. A small amount of EEG gel was carefully applied to each electrode using a syringe to avoid any bridging between neighboring electrodes. The impedance of the active electrodes was continuously monitored, ensuring it remained below 5 kΩ (Leong et al. 2018).

#### 2.5.1 ISF‐NF Training Protocol

Evidence from neuroimaging and neurophysiological studies demonstrates that ISF (frequency < 0.1 Hz) plays a profound role in defining intrinsic brain networks and modulating dynamic brain connections (Ploner et al. 2017; Raichle 2015). Moreover, the excitability of cortical networks is influenced by ISF and shows a strong correlation with the phase of higher‐frequency oscillations (Vanhatalo et al. 2005). Growing evidence suggests alterations in ISF and slow rhythmic fluctuations in individuals with chronic pain (Alshelh et al. 2018; Grooms et al. 2017). Additionally, neuroimaging studies indicate increased activity in pain‐related brain regions, such as the dACC and SSC, along with reduced ISF activity in the antinociceptive network, including the pgACC and its associated pathways (Grooms et al. 2017; Alshelh et al. 2016; Alshelh 2018; Zhou et al. 2018; Zhang et al. 2019; Majeed et al. 2011; Kucyi et al. 2013; Kucyi and Davis 2015; Di Pietro et al. 2020). Therefore, modulating ISF activity in these pain‐processing and mediating regions may influence cortical brain dynamics (Alshelh et al. 2016; Alshelh 2018; Zhou et al. 2018; Zhang et al. 2019; Majeed et al. 2011; Kucyi et al. 2013; Kucyi and Davis 2015; Di Pietro et al. 2020; Zhang et al. 2019). Thus, modulating ISF activity in regions involved in pain processing and mediation may have an impact on cortical brain dynamics.

**FIGURE 1:**
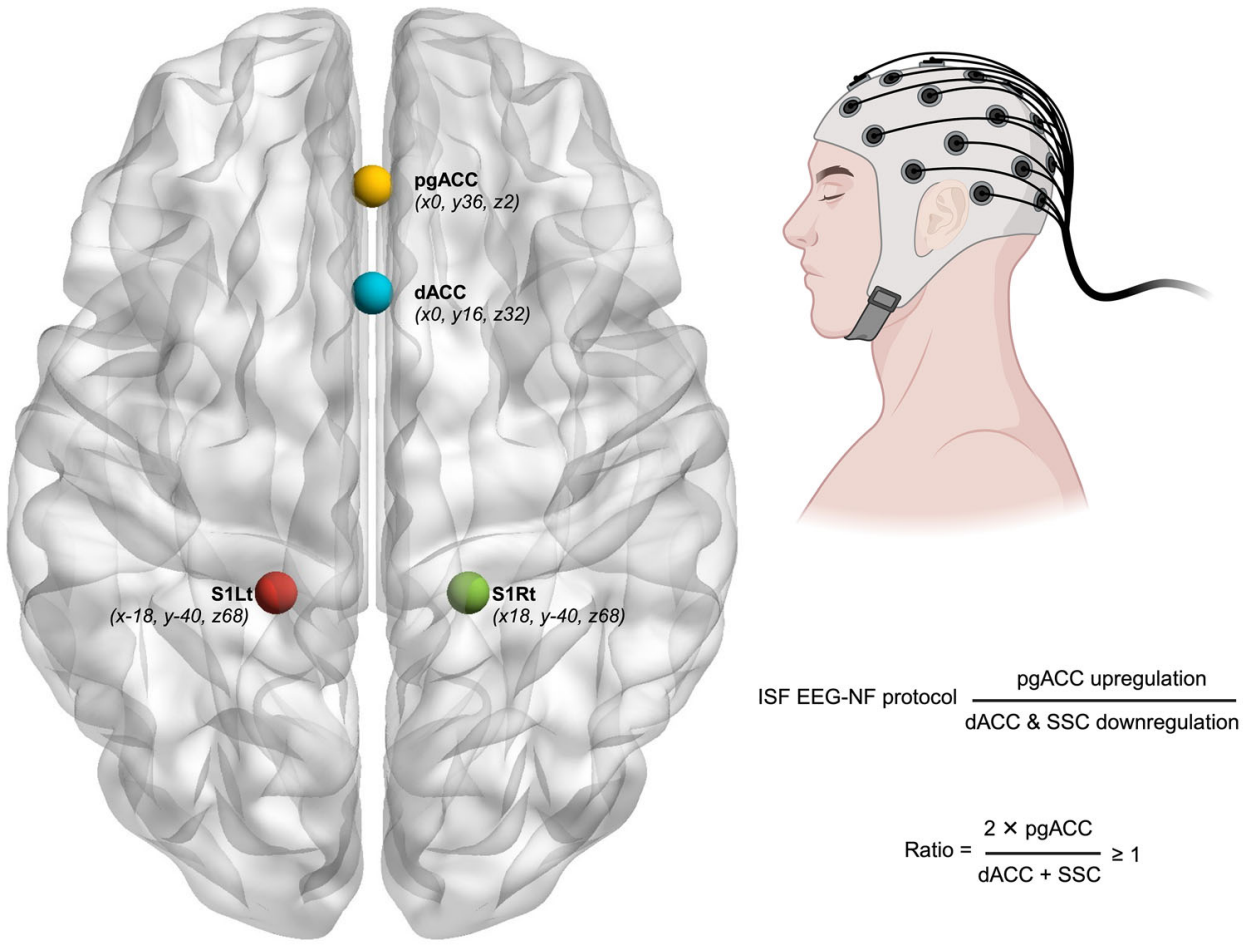
The regions of interest and ISF‐NF training directions. S1Lt: left somatosensory cortex; S1Rt: right somatosensory cortex; dACC: dorsal anterior cingulate cortex; pgACC: pregenual anterior cingulate cortex; The x, y, and z coordinates presented are the centroids of the target brain regions. The SSC encompassed Brodmann areas 1, 2, 3, and 5, as identified using the Montreal Neurological Institute (MNI) coordinate database (Fuchs et al. 2002; Lancaster et al. 2007). The dACC and pgACC were defined with the help of the Neurosynth meta‐analytic database (www.neurosynth.org) and previous literature (De Ridder et al. 2021; De Ridder et al. 2022; Vanneste and De Ridder 2021; De Ridder and Vanneste 2020; Mathew et al. 2022; Adhia et al. 2022; De Ridder et al. 2013). The image is created with https://www.nitrc.org/projects/bnv/ and www.biorender.com.

A training protocol for ISF‐NF was designed to optimize the equilibrium among the three cortical regions [SSC, dACC, and pgACC] (De Ridder et al. 2021; Vanneste and De Ridder 2021; De Ridder and Vanneste 2020; Mathew et al. 2022; Mathew et al. 2024b; Mathew et al. 2020). The selection of cortical brain regions and the design of the training protocol were informed by previous studies identifying an imbalance among the dACC, SSC, and pgACC (Vanneste and De Ridder 2021; De Ridder and Vanneste 2020). As illustrated in Figure 1, the ISF‐NF training simultaneously reduced ISF electrical activity in the SSC and dACC while enhancing activity in the pgACC.

The BrainAvatar software provided auditory feedback (reward) when the participant's brain activity reached the specified ISF (0.0–0.1 Hz) threshold at the designated regions of interest (ROI). The software computed the ratio in real‐time within the ISF band, delivering feedback when the ratio was ≥1, in accordance with the following equation (Mathew et al. 2022; Mathew et al. 2024b; Mathew et al. 2020):

$$\frac{2\times pgACC}{SSC+dACC}\geq 1$$

#### 2.5.2 Sham ISF‐NF Protocol

Participants in the sham‐NF group were prepared in the same way as the ISF‐NF group. However, the participants received sound feedback recorded from a healthy volunteer who underwent ISF‐NF training (yoked feedback/sham). We captured the feedback sound and replayed it to the sham group participants using Audacity software (Maheshkumar et al. 2016). The pre‐recorded signals were randomly chosen using the chit method from a collection of nine files.

Participants and the outcome assessor were blinded to group allocation and intervention. To assess the integrity of blinding, participants were asked at the end of the study which group they believed they were in. The methodology and findings related to blinding have been published elsewhere (Mathew et al. 2022).

### 2.6 Electroencephalography

Resting‐state EEG data (sampling rate of 500 Hz) were recorded (Mitsar EEG system with WinEEG software, Mitsar Co. Ltd, St. Petersburg, Russia) for ∼10 min with the participant's eyes closed at both T0 and T1 (Vanneste et al. 2018; Wyckoff et al. 2015; Grigolon et al. 2017; Yakovenko et al. 2021; Kim et al. 2015). EEG data were recorded using 21 channels positioned according to the standard 10–20 International system, referenced to linked ears, with impedances maintained below 5 kΩ (Khazi et al. 2012; Vanneste et al. 2018; Collura et al. 2025). The EEGLAB toolbox in MATLAB (R2020a) (Delorme and Makeig 2004; Delorme et al. 2011) was utilized to preprocess the EEG data (resampled to 128 Hz, bandpass filtered from 0.01 Hz to 44 Hz), and EEG artifacts, such as eye blinks, muscle activity, perspiration, and body movements, were eliminated using ICoN software (version 3) (Vanneste and De Ridder 2021; Adhia et al. 2022; Leong et al. 2018; Bersagliere et al. 2018).

#### 2.6.1 Granger Causality

Granger causality was employed to assess iCoh by calculating the partial coherence within a multivariate autoregressive model. This was followed by setting all non‐relevant associations to zero, leaving only the specific directional association of interest. Granger causality (EC) describes the strength of direct and directional causal information flow between two ROIs. iCoh detects both the peak frequency of information transfer from two distinct ROIs and the connection strength, which is inherently very high. The technical and mathematical models related to iCoh and Granger causality are available in previous publications (Pascual‐Marqui et al. 2014; Pascual‐Marqui et al. 2014). Using a multivariate autoregressive model, the iCoh methodology estimates the Granger causality (values range from 0 to 1). To estimate the information flow from one region to another, all other associations between the regions are set to zero, except the directional association of interest. In this way, iCoh is asymmetrical, i.e., for each pair of ROIs X and Y, the connectivity from X to Y is generally different than that from Y to X (Bosch‐Bayard et al. 2022).

The Granger causality between the targeted ROIs (pgACC, dACC, S1Lt, S1Rt) for the ISF frequency band (0.01 to 0.1 Hz) was calculated by means of the iCoh (Pascual‐Marqui et al. 2014). The iCoh was extracted from the pre‐processed EEG data using the open, exact low‐resolution electromagnetic tomography (eLORETA) method using the LORETA‐Key software (Collura et al. 2025; Pascual‐Marqui et al. 2011; Grech et al. 2008).

### 2.7 Data Analysis

All the statistical analyses were conducted using GraphPad Prism software version 9.1.0 for Windows (GraphPad Software, San Diego, CA, USA) (Motulsky 2003). A pre‐post analysis was conducted to compare the groups, calculating mean differences for all EEG measures from baseline (T0) to post‐intervention (T1). Larger mean differences reflected increased EC. The Shapiro–Wilk test and Q‐Q plots were used to assess the normality of the data for all variables. Given these findings, a non‐parametric Mann‐Whitney U test was employed to compare the distributions between the two groups. The p‐values were corrected for multiple comparisons using the Holm‐Šídák method, with a significance threshold set at ≤0.0042. Brain images were generated with BrainNet Viewer (https://www.nitrc.org/projects/bnv/) (Xia et al. 2013) and visualized using BioRender (https://www.biorender.com/).

## 3 Results

### 3.1 Demographics

A total of twenty‐one participants with knee OA were assessed at baseline and randomly assigned to either the ISF‐NF group (n = 11) or the sham‐NF group (n = 10). The active and sham groups had similar mean ages (62.3±8.5 and 61.0±6.7 years, respectively). Females comprised the majority in both groups (active: 64%; sham: 60%). Most participants identified as New Zealand European (Active: 91%; Sham: 90%), with a smaller proportion identifying as Australian (active: 9%) or Tongan (sham: 10%). The average pain score over the past three months was comparable between groups (active: 6.1±1.5; sham: 5.9±1.2). Right knee involvement was more common in both groups (active: 72.7%; sham: 70%) and the duration of knee pain was longer in the active group (5.3±4 years) compared to the sham group (2.6±2.3 years).

### 3.2 Granger Causality—iCoh

**FIGURE 2:**
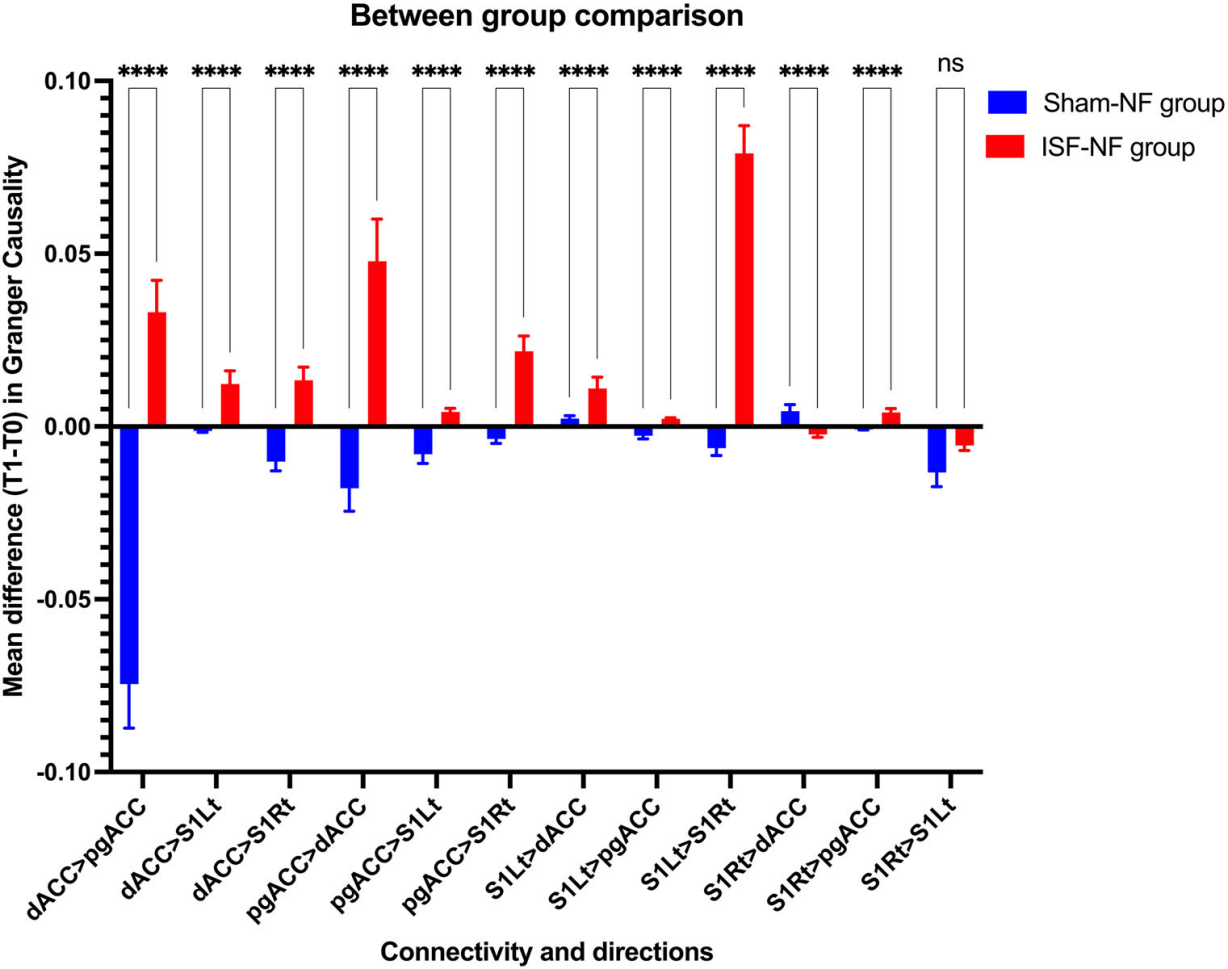
Results from the Mann–Whitney U tests between the ISF‐NF treatment groups and the Sham‐NF group. S1Lt: left somatosensory cortex; S1Rt: right somatosensory cortex; dACC: dorsal anterior cingulate cortex; pgACC: pregenual anterior cingulate cortex. P ≤ 0.0001 are denoted by (****).

**FIGURE 3:**
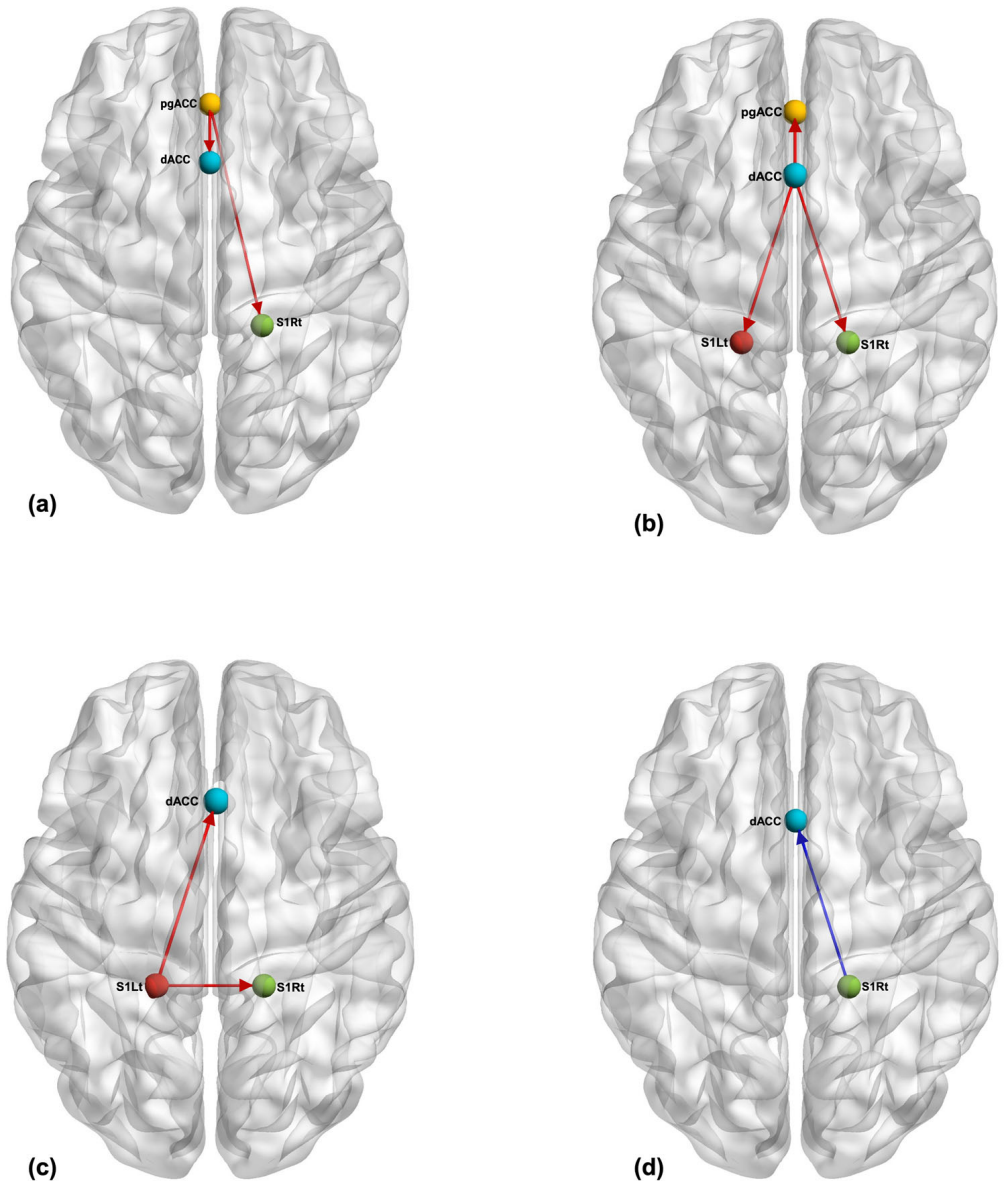
Results of the significant effective connectivity between the ROIs for the ISF‐NF group compared to the sham‐NF group. The red arrows indicate an increase in effective connectivity, and the blue arrow represents the reduction between the ROIs. S1Lt: left somatosensory cortex; S1Rt: right somatosensory cortex; dACC: dorsal anterior cingulate cortex; pgACC: pregenual anterior cingulate cortex. Created with https://www.nitrc.org/projects/bnv/ and www.biorender.com.

The iCoh was calculated for all the connections between the targeted ROIs in the ISF frequency bands. The ROIs include pgACC, SSC (left and right) and dACC. The Mann–Whitney U tests were conducted for these EC measures to compare the ISF‐NF treatment group with the Sham‐NF group individually, and significant connections are illustrated in Figure 2. Additionally, Figure 3 visually represents the EC for all the significant connections in the ISF‐NF group compared to the sham‐NF group. There was a significant increase in EC for (i) pgACC to dACC, (ii) pgACC to S1Rt, (iii) dACC to pgACC, (iv) dACC to S1Lt, (v) dACC to S1Rt, (vi) S1Lt to S1Rt, (vii) S1Lt to dACC, and (viii) decreased EC from S1Rt to dACC.

## 4 Discussion

This study sought to determine whether EEG‐NF training can alter EC between the targeted ROIs linked to pain‐mediating cortical brain regions in the ISF frequency band. The study highlighted improved EC between the key somatosensory, affective, and pain‐inhibitory cortical brain regions in the ISF frequency band. The study found a significant increase in EC in the following pathways: pgACC to dACC, pgACC to S1Rt, dACC to pgACC, dACC to S1Lt, dACC to S1Rt, S1Lt to S1Rt, and S1Lt to dACC. Additionally, there was a significant decrease in EC from S1Rt to dACC. Results from this study would contribute to the growing body of literature on the specificity of EEG‐NF by influencing the cortical neuronal oscillations, paving the way for future robust and novel EEG‐NF protocols for pain management.

Chronic pain can be accompanied by connectivity alterations in brain regions or associated networks linked to pain experience (Apkarian et al. 2011; Baliki et al. 2012; Bingel et al. 2006; Ohara et al. 2006; Napadow et al. 2010). The findings from our study indicate EC changes between the ROIs in the ISF band after NF training. Overall, the EC results of this study demonstrate improved communication from pgACC to dACC and pgACC to bilateral SSC in the ISF band compared to the sham‐NF group. This means there is an increased flow of information from pgACC to sensory and emotional cortices. Previous studies of chronic neuropathic pain have demonstrated reduced communication from pgACC to bilateral SSC and increased information transfer from bilateral SSC to pgACC for patients in comparison to controls (Vanneste and De Ridder 2021). This finding implies that the activity of the pgACC (key cortical anti‐nociceptive region) is inhibited by the increased information transfer from the SSC (sensory‐discriminative region). This information could indicate that ISF‐NF can modulate and influence communication between distant cortical regions in the same network.

Moreover, results from our previous study highlight increased EC from dACC to pgACC, inhibiting the activity of pgACC to communicate with dACC and SSC (De Ridder et al. 2021; Vanneste and De Ridder 2021; De Ridder and Vanneste 2020). Interestingly, the current study demonstrates a significant increase in the EC from pgACC to dACC in the ISF‐NF group post‐intervention. The study also observed increased EC from pgACC to the SSC. One element of the ISF‐NF ratio focused on enhancing the ISF activity of the pgACC, and we speculate that this training likely affected the functional communication between the pgACC and SSC. The study also found improved EC from S1Lt to S1Rt and S1Lt to dACC. The opposite pattern was observed from S1Rt to the dACC. The primary somatosensory cortex receives sensory information from the ventral posterolateral nucleus of the thalamus through the internal capsule, specifically from contralateral body regions. Interestingly, 72% of participants in the active group identified their right knee as the most painful joint. This finding indicates that somatosensory information is directed to and processed in the left SSC. Previous studies have confirmed reduced communication between the SSC and dACC, consequently amplifying the affective symptomatology of pain (Henderson et al. 2013; Vanneste and De Ridder 2021). Previous research has identified the dACC as a region involved in processing the unpleasant and emotional components of pain (Bushnell et al. 2013; Price 2000; Osaka et al. 2004; Rainville et al. 1997; Russo and Sheth 2015; Maeoka et al. 2012). This is a significant finding that reinforces the idea that the observed ISF effects between the SSC and dACC are probably influenced by the downregulation of both regions.

It is, however, worth mentioning that there is a paucity of EC data for chronic pain, and future trials need to confirm the effect of neuromodulatory intervention on EC changes in the studied population. The results of this study need to be interpreted with consideration of the study's limitations. We analyzed a small sample of knee OA individuals with a homogeneous sex and race distribution, which restricts the generalizability of the study findings. Also, our study did not include a full follow‐up assessment after weeks to months of the NF training, and we believe this is an important component that needs to be considered in future clinical trials investigating the effect of balance ISF‐NF for chronic pain as per the recommendations (Ros et al. 2020). Notwithstanding the relatively limited sample, this study provides an important foundation for future EEG‐NF studies to incorporate connectivity‐based analyses to understand the changes in cortical brain dynamics following the treatment. The study also highlights the potential of designing and implementing balance/ratio training NF protocols using different ROIs based on the clinical population and expected study outcomes. The balance training protocols used in this study can be efficient instead of training individual ROIs. However, future trials should investigate whether a balance/ratio training protocol is better than each ROI training in improving clinical outcomes. Similarly, it is also worth investigating the effect of ISF‐NF against the other existing NF protocols for chronic pain.

## 5 Conclusions

Balance ISF‐NF training can increase the EC between pain‐mediating cortical brain regions in the ISF band in individuals suffering from chronic knee OA pain. Caution is warranted in interpreting the observations since the study outcomes are based on exploratory analysis of a pilot and feasibility clinical trial. However, these observations suggest the need to explore the change in the EC‐mediates clinical outcomes following ISF‐NF training in a fully powered clinical trial. Moreover, there is an opportunity to develop newer EC‐based NF protocols and test for their clinical efficacy.

## Author Contributions

**Jerin Mathew**: conceptualization, data curation, formal analysis, investigation, methodology, project administration, software, validation, visualization, writing–original draft, writing–review and editing. **Divya Bharatkumar Adhia**: conceptualization, funding acquisition, investigation, methodology, resources, software, supervision, validation, visualization, writing–review and editing. **Mark Llewellyn Smith**: methodology, software, writing–review and editing. **Dirk De Ridder**: conceptualization, methodology, resources, supervision, validation, visualization, writing–review and editing. **Ramakrishnan Mani**: conceptualization, data curation, funding acquisition, investigation, methodology, resources, supervision, validation, visualization, writing–review and editing.

## Ethics Statement

This study received ethical approval from the Health & Disability Ethics Committee (HDEC), New Zealand (19CEN182), and was registered with the Australian New Zealand Clinical Trials Registry (ACTRN12620000273987).

## Conflicts of Interest

The authors report no conflicts of interest.

### Peer Review

The peer review history for this article is available at https://publons.com/publon/10.1002/brb3.70541

## Data Availability

The data that support the findings of this study are available on reasonable request from the corresponding author. The data are not publicly available due to privacy or ethical restrictions.

## References

[brb370541-bib-0001] Adhia, D. B. , R. Mani , J. Mathew , et al. 2023. “Exploring Electroencephalographic Infraslow Neurofeedback Treatment for Chronic Low Back Pain: A Double‐Blinded Safety and Feasibility Randomized Placebo‐Controlled Trial.” Scientific Reports 13, no. 1: 1177. 36670176 10.1038/s41598-023-28344-2 PMC9860016

[brb370541-bib-0002] Adhia, D. B. , R. Mani , J. N. J. Reynolds , M. Hall , S. Vanneste , and D. De Ridder . 2023. “High‐Definition Transcranial Infraslow Pink‐Noise Stimulation Can Influence Functional and Effective Cortical Connectivity in Individuals with Chronic Low Back Pain: A Pilot Randomized Placebo‐Controlled Study.” Neuromodulation 26, no. 4: 788–800. 36272898 10.1016/j.neurom.2022.08.450

[brb370541-bib-0003] Adhia, D. B. , R. Mani , P. R. Turner , S. Vanneste , and D. De Ridder . 2022. “Infraslow Neurofeedback Training Alters Effective Connectivity in Individuals With Chronic Low Back Pain: A Secondary Analysis of a Pilot Randomized Placebo‐Controlled Study.” Brain Sciences 12, no. 11: 1514. 36358440 10.3390/brainsci12111514 PMC9688799

[brb370541-bib-0004] Alshelh, Z. 2018. “Infra‐Slow Oscillations in Chronic Orofacial Neuropathic Pain and the Effects of Palmitoylethanolamide.” PhD thesis, University of Sydney.

[brb370541-bib-0005] Alshelh, Z. , F. Di Pietro , A. M. Youssef , et al. 2016. “Chronic Neuropathic Pain: It's About the Rhythm.” The Journal of Neuroscience 36, no. 3: 1008–1018. 26791228 10.1523/JNEUROSCI.2768-15.2016 PMC6602000

[brb370541-bib-0006] Alshelh, Z. , K. K. Marciszewski , R. Akhter , et al. 2018. “Disruption of Default Mode Network Dynamics in Acute and Chronic Pain States.” NeuroImage: Clinical 17: 222–231. 29159039 10.1016/j.nicl.2017.10.019 PMC5683191

[brb370541-bib-0007] Apkarian, V. A. , J. A. Hashmi , and M. N. Baliki . 2011. “Pain and the Brain: Specificity and Plasticity of the Brain in Clinical Chronic Pain.” Pain 152: no. 3 Suppl: S49–S64. 21146929 10.1016/j.pain.2010.11.010 PMC3045648

[brb370541-bib-0008] Baliki, M. N. , B. Petre , S. Torbey , et al. 2012. “Corticostriatal Functional Connectivity Predicts Transition to Chronic Back Pain.” Nature Neuroscience 15, no. 8: 1117–1119. 22751038 10.1038/nn.3153 PMC3411898

[brb370541-bib-0010] Barroso, J. , K. Wakaizumi , A. M. Reis , et al. 2021. “Reorganization of Functional Brain Network Architecture in Chronic Osteoarthritis Pain.” Human Brain Mapping 42, no. 4: 1206–1222. 33210801 10.1002/hbm.25287 PMC7856636

[brb370541-bib-0011] Bersagliere, A. , R. D. Pascual‐Marqui , L. Tarokh , and P. Achermann . 2018. “Mapping Slow Waves by EEG Topography and Source Localization: Effects of Sleep Deprivation.” Brain Topography 31, no. 2: 257–269. 28983703 10.1007/s10548-017-0595-6

[brb370541-bib-0012] Bingel, U. , J. Lorenz , E. Schoell , C. Weiller , and C. Büchel . 2006. “Mechanisms of Placebo Analgesia: rACC Recruitment of a Subcortical Antinociceptive Network.” Pain 120, no. 1: 8–15. 16364549 10.1016/j.pain.2005.08.027

[brb370541-bib-0013] Bosch‐Bayard, J. , R. J. Biscay , T. Fernandez , et al. 2022. “EEG Effective Connectivity During the First Year of Life Mirrors Brain Synaptogenesis, Myelination, and Early Right Hemisphere Predominance.” Neuroimage 252: 119035. 35218932 10.1016/j.neuroimage.2022.119035

[brb370541-bib-0014] Brandl, F. , B. Weise , S. Mulej Bratec , et al. 2022. “Common and Specific Large‐Scale Brain Changes in Major Depressive Disorder, Anxiety Disorders, and Chronic Pain: A Transdiagnostic Multimodal Meta‐Analysis of Structural and Functional MRI Studies.” Neuropsychopharmacology 47: 1071–1080. 35058584 10.1038/s41386-022-01271-y PMC8938548

[brb370541-bib-0015] Bushnell, M. C. , M. Čeko , and L. A. Low . 2013. “Cognitive and Emotional Control of Pain and Its Disruption in Chronic Pain.” Nature Reviews Neuroscience 14, no. 7: 502–511. 23719569 10.1038/nrn3516 PMC4465351

[brb370541-bib-0016] Bushnell, M. C. , G. H. Duncan , R. K. Hofbauer , et al. 1999. “Pain Perception: Is There a Role for Primary Somatosensory Cortex?” Proceedings of the National Academy of Sciences 96, no. 14: 7705–7709. 10.1073/pnas.96.14.7705 PMC33605 10393884

[brb370541-bib-0017] Cao, J. , Y. Zhao , X. Shan , et al. 2022. “Brain Functional and Effective Connectivity Based on Electroencephalography Recordings: A Review.” Human Brain Mapping 43, no. 2: 860–879. 34668603 10.1002/hbm.25683 PMC8720201

[brb370541-bib-0018] Collura, T. , D. Cantor , D. Chartier , et al. 2025. “International QEEG Certification Board Guideline Minimum Technical Requirements for Performing Clinical Quantitative Electroencephalography.” Clinical EEG and Neuroscience 15500594241308654. 10.1177/15500594241308654. 39901446

[brb370541-bib-0020] Craig, A. D. 2002. “How Do You Feel? Interoception: The Sense of the Physiological Condition of the Body.” Nature Reviews Neuroscience 3, no. 8: 655–666. 12154366 10.1038/nrn894

[brb370541-bib-0022] Delorme, A. , and S. Makeig . 2004. “EEGLAB: An Open Source Toolbox for Analysis of Single‐Trial EEG Dynamics Including Independent Component Analysis.” Journal of Neuroscience Methods 134, no. 1: 9–21. 15102499 10.1016/j.jneumeth.2003.10.009

[brb370541-bib-0023] Delorme, A. , T. Mullen , C. Kothe , et al. 2011. “EEGLAB, SIFT, NFT, BCILAB, and ERICA: New Tools for Advanced EEG Processing.” Computational Intelligence and Neuroscience 2011: 1–12. 21687590 10.1155/2011/130714 PMC3114412

[brb370541-bib-0024] De Ridder, D. , D. Adhia , and S. Vanneste . 2021. “The Anatomy of Pain and Suffering in the Brain and Its Clinical Implications.” Neuroscience & Biobehavioral Reviews 130: 125–146. 34411559 10.1016/j.neubiorev.2021.08.013

[brb370541-bib-0025] De Ridder, D. , and S. Vanneste . 2020. “The Bayesian Brain in Imbalance: Medial, Lateral and Descending Pathways in Tinnitus and Pain: A perspective.” in Progress in Brain Research. Elsevier. 10.1016/bs.pbr.2020.07.012 33931186

[brb370541-bib-0026] De Ridder, D. , S. Vanneste , M. Smith , and D. Adhia . 2022. “Pain and the Triple Network Model.” Frontiers in Neurology 13: 757241. 35321511 10.3389/fneur.2022.757241 PMC8934778

[brb370541-bib-0027] De Ridder, D. , J. Verplaetse , and S. Vanneste . 2013. “The Predictive Brain and the “Free Will” Illusion.” Frontiers in Psychology 4: 131. 23641219 10.3389/fpsyg.2013.00131 PMC3639403

[brb370541-bib-0028] Di Pietro, F. , B. Lee , and L. A. Henderson . 2020. “Altered Resting Activity Patterns and Connectivity in Individuals With Complex Regional Pain Syndrome.” Human Brain Mapping 41, no. 13: 3781–3793. 32510695 10.1002/hbm.25087 PMC7416050

[brb370541-bib-0029] Eldridge, S. M. , C. L. Chan , M. J. Campbell , et al. 2016. “CONSORT 2010 Statement: Extension to Randomised Pilot and Feasibility Trials.” Bmj 355: i5239. 27777223 10.1136/bmj.i5239 PMC5076380

[brb370541-bib-0030] Enriquez‐Geppert, S. , R. J. Huster , and C. S. Herrmann . 2017. “EEG‐Neurofeedback as a Tool to Modulate Cognition and Behavior: A Review Tutorial.” Frontiers in Human Neuroscience 11: 51. 28275344 10.3389/fnhum.2017.00051 PMC5319996

[brb370541-bib-0031] Fallon, N. , Y. Chiu , T. Nurmikko , and A. Stancak . 2016. “Functional Connectivity With the Default Mode Network Is Altered in Fibromyalgia Patients.” PLoS ONE 11, no. 7: e0159198. 27442504 10.1371/journal.pone.0159198 PMC4956096

[brb370541-bib-0032] Ferdek, M. A. , J. M. Oosterman , A. K. Adamczyk , et al. 2019. “Effective Connectivity of Beta Oscillations in Endometriosis‐Related Chronic Pain During Rest and Pain‐Related Mental Imagery.” The Journal of Pain 20, no. 12: 1446–1458. 31152855 10.1016/j.jpain.2019.05.011

[brb370541-bib-0033] Fields, H. 2004. “State‐Dependent Opioid Control of Pain.” Nature Reviews Neuroscience 5, no. 7: 565–575. 15208698 10.1038/nrn1431

[brb370541-bib-0034] Friston, K. J. 2011. “Functional and Effective Connectivity: A Review.” Brain Connectivity 1, no. 1: 13–36. 22432952 10.1089/brain.2011.0008

[brb370541-bib-0035] Fuchs, M. , J. Kastner , M. Wagner , S. Hawes , and J. S. Ebersole . 2002. “A Standardized Boundary Element Method Volume Conductor Model.” Clinical Neurophysiology 113, no. 5: 702–712. 11976050 10.1016/s1388-2457(02)00030-5

[brb370541-bib-0037] Gingold, S. I. , J. D. Greenspan , and A. V. Apkarian . 1991. “Anatomic Evidence of Nociceptive Inputs to Primary Somatosensory Cortex—Relationship Between Spinothalamic Terminals and Thalamocortical Cells in Squirrel‐Monkeys.” Journal of Comparative Neurology 308, no. 3: 467–490. 1865012 10.1002/cne.903080312

[brb370541-bib-0038] Grech, R. , T. Cassar , J. Muscat , et al. 2008. “Review on Solving the Inverse Problem in EEG Source Analysis.” Journal of Neuroengineering and Rehabilitation 5, no. 1: 1–33. 18990257 10.1186/1743-0003-5-25 PMC2605581

[brb370541-bib-0039] Grigolon, R. , Q. Cordeiro , and A. Trevizol . 2017. “Transcutaneous Auricular Vagus Nerve Stimulation for Food Craving: Study Protocol for a Phase II Randomized, Sham‐Controlled Clinical Trial.” Asia Pacific Journal of Clinical Trials: Nervous System Diseases 2, no. 3: 91–91.

[brb370541-bib-0040] Grooms, J. K. , G. J. Thompson , W.‐J.u Pan , et al. 2017. “Infraslow Electroencephalographic and Dynamic Resting State Network Activity.” Brain Connectivity 7, no. 5: 265–280. 28462586 10.1089/brain.2017.0492 PMC5510044

[brb370541-bib-0041] Henderson, L. A. , C. C. Peck , E. T. Petersen , et al. 2013. “Chronic Pain: Lost Inhibition?” Journal of Neuroscience 33, no. 17: 7574–7582. 23616562 10.1523/JNEUROSCI.0174-13.2013 PMC6619566

[brb370541-bib-0042] Hesam‐Shariati, N. , W. J. Chang , M. A. Wewege , et al. 2021. “The Analgesic Effect of Electroencephalographic Neurofeedback for People With Chronic Pain: A Systematic Review and Meta‐Analysis.” European Journal of Neurology 29, no. 3: 921–936. 34813662 10.1111/ene.15189

[brb370541-bib-0043] Hesam‐Shariati, N. , W.‐J.u Chang , M. A. Wewege , et al. 2022. “The Analgesic Effect of Electroencephalographic Neurofeedback for People With Chronic Pain: A Systematic Review and Meta‐Analysis.” European Journal of Neurology 29, no. 3: 921–936. 34813662 10.1111/ene.15189

[brb370541-bib-0044] Hoffmann, T. C. , P. P. Glasziou , I. Boutron , et al. 2014. “Better Reporting of Interventions: Template for Intervention Description and Replication (TIDieR) Checklist and Guide.” Bmj 348: g1687. 24609605 10.1136/bmj.g1687

[brb370541-bib-0045] Kaplan, C. M. , A. Schrepf , D. Vatansever , et al. 2019. “Functional and Neurochemical Disruptions of Brain Hub Topology in Chronic Pain.” Pain 160, no. 4: 973–983. 30763287 10.1097/j.pain.0000000000001480 PMC6424595

[brb370541-bib-0046] Khazi, M. , A. Kumar , and M. Vidya . 2012. “Analysis of EEG Using 10: 20 Electrode System.” International Journal of Innovative Research in Science, Engineering and Technology 1, no. 2: 185–191.

[brb370541-bib-0047] Kim, D. , C. Yeon , E. Chung , and K. Kim . 2015. “Experimental Validation of Mouse EEG Sensor Through the Analysis of Visually Evoked Potential Elicited by Successive Flash Stimuli.” in 2015 IEEE SENSORS. IEEE.

[brb370541-bib-0048] Kucyi, A. , and K. D. Davis . 2015. “The Dynamic Pain Connectome.” Trends in Neurosciences 38, no. 2: 86–95. 25541287 10.1016/j.tins.2014.11.006

[brb370541-bib-0049] Kucyi, A. , T. V. Salomons , and K. D. Davis . 2013. “Mind Wandering Away From Pain Dynamically Engages Antinociceptive and Default Mode Brain Networks.” PNAS 110, no. 46: 18692–18697. 24167282 10.1073/pnas.1312902110 PMC3832014

[brb370541-bib-0050] Kwon, M. , M. Altin , H. Duenas , and L. Alev . 2014. “The Role of Descending Inhibitory Pathways on Chronic Pain Modulation and Clinical Implications.” Pain Practice 14, no. 7: 656–667. 24256177 10.1111/papr.12145

[brb370541-bib-0051] Lancaster, J. L. , D. Tordesillas‐Gutiérrez , M. Martinez , et al. 2007. “Bias Between MNI and Talairach Coordinates Analyzed Using the ICBM‐152 Brain Template.” Human Brain Mapping 28, no. 11: 1194–1205. 17266101 10.1002/hbm.20345 PMC6871323

[brb370541-bib-0052] Leong, S. L. , S. Vanneste , J. Lim , M. Smith , P. Manning , and D. De Ridder . 2018. “A Randomised, Double‐Blind, Placebo‐Controlled Parallel Trial of Closed‐Loop Infraslow Brain Training in Food Addiction.” Scientific Reports 8, no. 1: 11659. 30076365 10.1038/s41598-018-30181-7 PMC6076277

[brb370541-bib-0053] Lewis, G. N. , R. S. Parker , S. Sharma , D. A. Rice , and P. J. Mcnair . 2018. “Structural Brain Alterations Before and After Total Knee Arthroplasty: A Longitudinal Assessment.” Pain Medicine 19, no. 11: 2166–2176. 29917139 10.1093/pm/pny108

[brb370541-bib-0054] Maeoka, H. , A. Matsuo , M. Hiyamizu , S. Morioka , and H. Ando . 2012. “Influence of Transcranial Direct Current Stimulation of the Dorsolateral Prefrontal Cortex on Pain Related Emotions: A Study Using Electroencephalographic Power Spectrum Analysis.” Neuroscience Letters 512, no. 1: 12–16. 22326385 10.1016/j.neulet.2012.01.037

[brb370541-bib-0055] Maheshkumar, K. , K. Dilara , K. Maruthy , and L. Sundareswaren . 2016. “Validation of PC‐Based Sound Card With Biopac for Digitalization of ECG Recording in Short‐Term HRV Analysis.” North American Journal of Medicine and Science 8, no. 7: 307. 10.4103/1947-2714.187150 PMC4982360 27583239

[brb370541-bib-0056] Majeed, W. , M. Magnuson , W. Hasenkamp , et al. 2011. “Spatiotemporal Dynamics of Low Frequency BOLD Fluctuations in Rats and Humans.” Neuroimage 54, no. 2: 1140–1150. 20728554 10.1016/j.neuroimage.2010.08.030 PMC2997178

[brb370541-bib-0057] Mao, C. P. , H. J. Yang , Q. J. Zhang , Q. X. Yang , and X. H. Li . 2022. “Altered Effective Connectivity Within the Cingulo‐Frontal‐Parietal Cognitive Attention Networks in Chronic Low Back Pain: A Dynamic Causal Modeling Study.” Brain Imaging and Behavior 16, no. 4: 1516–1527. 35080703 10.1007/s11682-021-00623-4

[brb370541-bib-0058] Marzbani, H. , H. R. Marateb , and M. Mansourian . 2016. “Neurofeedback: A Comprehensive Review on System Design, Methodology and Clinical Applications.” Basic and Clinical Neuroscience 7, no. 2: 143–158. 27303609 10.15412/J.BCN.03070208 PMC4892319

[brb370541-bib-0059] Mathew, J. , D. Adhia , M. Smith , D. De Ridder , and R. Mani . 2020. “Protocol for a Pilot Randomized Sham‐Controlled Clinical Trial Evaluating the Feasibility, Safety, and Acceptability of Infraslow Electroencephalography Neurofeedback Training on Experimental and Clinical Pain Outcomes in People With Chronic Painful Knee.” NeuroRegulation 7, no. 1: 30–44.

[brb370541-bib-0061] Mathew, J. , D. B. Adhia , M. Hall , D. De Ridder , and R. Mani . 2024a. “EEG‐Based Cortical Alterations in Individuals with Chronic Knee Pain Secondary to Osteoarthritis: A Cross‐Sectional Investigation.” The Journal of Pain 25, no. 5: 104429. 37989404 10.1016/j.jpain.2023.11.012

[brb370541-bib-0063] Mathew, J. , D. B. Adhia , M. L. Smith , D. De Ridder , and R. Mani . 2022. “Source Localized Infraslow Neurofeedback Training in People With Chronic Painful Knee Osteoarthritis: A Randomized, Double‐Blind, Sham‐Controlled Feasibility Clinical Trial.” Frontiers in Neuroscience 16: 00. 10.3389/fnins.2022.899772 PMC9366917 35968375

[brb370541-bib-0064] Mathew, J. , D. B. Adhia , M. L. Smith , D. De Ridder , and R. Mani . 2024b. “Closed‐Loop Infraslow Brain–Computer Interface Can Modulate Cortical Activity and Connectivity in Individuals with Chronic Painful Knee Osteoarthritis: A Secondary Analysis of a Randomized Placebo‐Controlled Clinical Trial.” Clinical EEG and Neuroscience 56, no. 2: 165–180. 10.1177/15500594241264892. 39056313 PMC11800731

[brb370541-bib-0111] Mathew, J. 2025. “Neurofeedback‐Based Brain‐Computer Interface for Pain Management: A Research Perspective.” New Zealand Journal of Physiotherapy 53, no. 1: 4–6. 10.15619/nzjp.v53i1.479.

[brb370541-bib-0066] Meeker, T. J. , R. Jupudi , F. A. Lenz , and J. D. Greenspan . 2020. “New Developments in Non‐Invasive Brain Stimulation in Chronic Pain.” Current Physical Medicine and Rehabilitation Reports 8, no. 3: 280–292. 33473332 10.1007/s40141-020-00260-w PMC7814313

[brb370541-bib-0068] Motoyama, Y. , Y. Oshiro , Y. Takao , et al. 2019. “Resting‐State Brain Functional Connectivity in Patients With Chronic Pain Who Responded to Subanesthetic‐Dose Ketamine.” Scientific Reports 9, no. 1: 12912. 31501482 10.1038/s41598-019-49360-1 PMC6733873

[brb370541-bib-0069] Motulsky, H. 2003. Prism 4 Statistics Guide—Statistical Analyses for Laboratory and Clinical Researchers. GraphPad Software Inc., 122–126.

[brb370541-bib-0070] Napadow, V. , L. LaCount , K. Park , S. As‐Sanie , D. J. Clauw , and R. E. Harris . 2010. “Intrinsic Brain Connectivity in Fibromyalgia Is Associated With Chronic Pain Intensity.” Arthritis & Rheumatism 62, no. 8: 2545–2555. 20506181 10.1002/art.27497 PMC2921024

[brb370541-bib-0071] Necka, E. A. , I.n‐S. Lee , A. Kucyi , J. C. Cheng , Q. Yu , and L. Y. Atlas . 2019. “Applications of Dynamic Functional Connectivity to Pain and Its Modulation.” Pain Reports 4, no. 4: e752. 31579848 10.1097/PR9.0000000000000752 PMC6728009

[brb370541-bib-0072] Ohara, S. , N. E. Crone , N. Weiss , and F. A. Lenz . 2006. “Analysis of Synchrony Demonstrates ‘Pain Networks’ Defined by Rapidly Switching, Task‐Specific, Functional Connectivity Between Pain‐Related Cortical Structures.” Pain 123, no. 3: 244–253. 16563627 10.1016/j.pain.2006.02.012

[brb370541-bib-0074] Osaka, N. , M. Osaka , M. Morishita , H. Kondo , and H. Fukuyama . 2004. “A Word Expressing Affective Pain Activates the Anterior Cingulate Cortex in the Human Brain: An fMRI Study.” Behavioural Brain Research 153, no. 1: 123–127. 15219713 10.1016/j.bbr.2003.11.013

[brb370541-bib-0075] Ossipov, M. H. , K. Morimura , and F. Porreca . 2014. “Descending Pain Modulation and Chronification of Pain.” Current Opinion in Supportive and Palliative Care 8, no. 2: 143–151. 24752199 10.1097/SPC.0000000000000055 PMC4301419

[brb370541-bib-0076] Pascual‐Marqui, R. , R. J. Biscay , J. Bosch‐Bayard , et al. 2014. “Isolated Effective Coherence (iCoh): Causal Information Flow Excluding Indirect Paths.” Preprint, arXiv:1402.4887, May 12. 10.3389/fnhum.2014.00448 PMC4064566 24999323

[brb370541-bib-0077] Pascual‐Marqui, R. D. , R. J. Biscay , J. Bosch‐Bayard , et al. 2014. “Assessing Direct Paths of Intracortical Causal Information Flow of Oscillatory Activity With the Isolated Effective Coherence (iCoh).” Frontiers in Human Neuroscience 8: 448. 24999323 10.3389/fnhum.2014.00448 PMC4064566

[brb370541-bib-0078] Pascual‐Marqui, R. D. , D. Lehmann , M. Koukkou , et al. 2011. “Assessing Interactions in the Brain With Exact Low‐Resolution Electromagnetic Tomography.” Philosophical Transactions of the Royal Society A: Mathematical, Physical and Engineering Sciences 369, no. 1952: 3768–3784. 10.1098/rsta.2011.0081 21893527

[brb370541-bib-0080] Ploner, M. , C. Sorg , and J. Gross . 2017. “Brain Rhythms of Pain.” Trends in Cognitive Sciences 21, no. 2: 100–110. 28025007 10.1016/j.tics.2016.12.001 PMC5374269

[brb370541-bib-0081] Price, D. D. 2000. “Psychological and Neural Mechanisms of the Affective Dimension of Pain.” Science 288, no. 5472: 1769–1772. 10846154 10.1126/science.288.5472.1769

[brb370541-bib-0082] Raichle, M. E. 2015. “The Restless Brain: How Intrinsic Activity Organizes Brain Function.” Philosophical Transactions of the Royal Society of London. Series B, Biological Sciences 370, no. 1668: 20140172. 25823869 10.1098/rstb.2014.0172 PMC4387513

[brb370541-bib-0083] Rainville, P. 2002. “Brain Mechanisms of Pain Affect and Pain Modulation.” Current Opinion in Neurobiology 12, no. 2: 195–204. 12015237 10.1016/s0959-4388(02)00313-6

[brb370541-bib-0084] Rainville, P. , G. H. Duncan , D. D. Price , B. Carrier , and M. C. Bushnell . 1997. “Pain Affect Encoded in Human Anterior Cingulate but Not Somatosensory Cortex.” Science 277, no. 5328: 968–971. 9252330 10.1126/science.277.5328.968

[brb370541-bib-0085] Ros, T. , S. Enriquez‐Geppert , V. Zotev , et al. 2020. Consensus on the Reporting and Experimental Design of Clinical and Cognitive‐Behavioural Neurofeedback Studies (CRED‐nf checklist). Oxford University Press. 10.1093/brain/awaa009 PMC7296848 32176800

[brb370541-bib-0086] Roy, R. , R. De La Vega , M. P. Jensen , and J. Miró . 2020. “Neurofeedback for Pain Management: A Systematic Review.” Frontiers in neuroscience 14: 671. 32765208 10.3389/fnins.2020.00671 PMC7378966

[brb370541-bib-0087] Russo, J. F. , and S. A. Sheth . 2015. “Deep Brain Stimulation of the Dorsal Anterior Cingulate Cortex for the Treatment of Chronic Neuropathic Pain.” Neurosurgical Focus 38, no. 6: E11. 10.3171/2015.3.FOCUS1543 26030699

[brb370541-bib-0088] Sitaram, R. , T. Ros , L. Stoeckel , et al. 2017. “Closed‐Loop Brain Training: The Science of Neurofeedback.” Nature Reviews Neuroscience 18, no. 2: 86–100. 28003656 10.1038/nrn.2016.164

[brb370541-bib-0089] Spisak, T. , B. Kincses , F. Schlitt , et al. 2020. “Pain‐Free Resting‐State Functional Brain Connectivity Predicts Individual Pain Sensitivity.” Nature Communications 11, no. 1: 187. 10.1038/s41467-019-13785-z PMC6954277 31924769

[brb370541-bib-0090] Spisák, T. , Z. Pozsgay , C. Aranyi , et al. 2017. “Central Sensitization‐Related Changes of Effective and Functional Connectivity in the Rat Inflammatory Trigeminal Pain Model.” Neuroscience 344: 133–147. 28003158 10.1016/j.neuroscience.2016.12.018

[brb370541-bib-0092] Thorp, S. L. , T. Suchy , N. Vadivelu , E. M. Helander , R. D. Urman , and A. D. Kaye . 2018. “Functional Connectivity Alterations: Novel Therapy and Future Implications in Chronic Pain Management.” Pain Physician 21, no. 3: E207–E214. 29871376

[brb370541-bib-0094] Vanhatalo, S. , J. Voipio , and K. Kaila . 2005. “Full‐Band EEG (FbEEG): An Emerging Standard in Electroencephalography.” Clinical Neurophysiology 116, no. 1: 1–8. 15589176 10.1016/j.clinph.2004.09.015

[brb370541-bib-0095] Vanneste, S. , and D. De Ridder . 2021. “Chronic Pain as a Brain Imbalance Between Pain Input and Pain Suppression.” Brain Communications 3, no. 1: fcab014. 33758824 10.1093/braincomms/fcab014 PMC7966784

[brb370541-bib-0096] Vanneste, S. , K. Joos , J. Ost , and D. De Ridder . 2018. “Influencing Connectivity and Cross‐Frequency Coupling by Real‐Time Source Localized Neurofeedback of the Posterior Cingulate Cortex Reduces Tinnitus Related Distress.” Neurobiology of Stress 8: 211–224. 29888315 10.1016/j.ynstr.2016.11.003 PMC5991329

[brb370541-bib-0097] Vanneste, S. , J. Ost , T. Van Havenbergh , and D. De Ridder . 2017. “Resting State Electrical Brain Activity and Connectivity in Fibromyalgia.” PLoS ONE 12, no. 6: e0178516. 28650974 10.1371/journal.pone.0178516 PMC5484465

[brb370541-bib-0098] Vanneste, S. , J. J. Song , and D. De Ridder . 2018. “Thalamocortical Dysrhythmia Detected by Machine Learning.” Nature Communications 9, no. 1: 1103. 10.1038/s41467-018-02820-0 PMC5856824 29549239

[brb370541-bib-0099] Vogt, B. A. , and R. W. Sikes . 2000. “The Medial Pain System, Cingulate Cortex, and Parallel Processing of Nociceptive Information.” Progress in Brain Research 122: 223–235. 10737061 10.1016/s0079-6123(08)62141-x

[brb370541-bib-0100] Wu, G. , E. Tagliazucchi , D. R. Chialvo , and D. Marinazzo . 2013. Point‐Process Deconvolution of fMRI Reveals Effective Connectivity Alterations in Chronic Pain Patients. Preprint, arXiv:1310.8357, October 31.

[brb370541-bib-0101] Wyckoff, S. N. , L. H. Sherlin , N. L. Ford , and D. Dalke . 2015. “Validation of a Wireless Dry Electrode System for Electroencephalography.” Journal of Neuroengineering and Rehabilitation 12, no. 1: 1–9. 26520574 10.1186/s12984-015-0089-2 PMC4628242

[brb370541-bib-0102] Xia, M. , J. Wang , and Y. He . 2013. “BrainNet Viewer: A Network Visualization Tool for human Brain Connectomics.” PLoS ONE 8, no. 7: e68910. 23861951 10.1371/journal.pone.0068910 PMC3701683

[brb370541-bib-0104] Yakovenko, E. A. , A. V. Rem , S. Y.u. Surushkina , and L. S. Chutko . 2021. “Electroencephalographic Signs of Emotional Burnout Syndrome.” Neuroscience and Behavioral Physiology 51, no. 2: 155–157.

[brb370541-bib-0106] Yang, J. M. , C. C. Li , Y. Wang , et al. 2024. “Transcranial Direct Current Stimulation for Knee Osteoarthritis: A Systematic Review and Meta‐Analysis of Randomized Controlled Trials.” Arthritis Care & Research 76, no. 3: 376–384. 37779486 10.1002/acr.25249

[brb370541-bib-0107] Yang, S. , and M. C. Chang . 2019. “Chronic Pain: Structural and Functional Changes in Brain Structures and Associated Negative Affective States.” International Journal of Molecular Sciences 20, no. 13: 3130. 31248061 10.3390/ijms20133130 PMC6650904

[brb370541-bib-0108] Zhang, B. , M. Jung , Y. Tu , et al. 2019. “Identifying Brain Regions Associated With the Neuropathology of Chronic Low Back Pain: A Resting‐State Amplitude of Low‐Frequency Fluctuation Study.” British Journal of Anaesthesia 123, no. 2: e303–e311. 30948036 10.1016/j.bja.2019.02.021 PMC6676015

[brb370541-bib-0109] Zhang, Y. , Z. Mao , L. Pan , et al. 2019. “Frequency‐Specific Alterations in Cortical Rhythms and Functional Connectivity in Trigeminal Neuralgia.” Brain Imaging and Behavior 13, no. 6: 1497–1509. 31209834 10.1007/s11682-019-00105-8

[brb370541-bib-0110] Zhou, F. , L. Gu , S. Hong , et al. 2018. “Altered Low‐Frequency Oscillation Amplitude of Resting State‐fMRI in Patients With Discogenic Low‐Back and Leg Pain.” Journal of Pain Research 11: 165–176. 29386913 10.2147/JPR.S151562 PMC5767087

